# How to Walk and Stand

**Published:** 1871-01

**Authors:** 


*How to Walk and Stand.*—We have the following account of a lecture on this subject, by Prof. Burt G. Wilder, of Cornell University. The very position of the head upon the spinal column of mankind gives to him, and him alone, power to elevate his eyes, and which man would not be endowed with did he move forward and backward upon all fours. We have the power of grasping with the hands, and of touching every part of the body with the fingers of one and the other hand, which typifies and represents in the bodies that freedom of will which man possesses beyond all other animals: and, finally, we walk erect, and there must be certainly something in this beyond the mere fact that man's body is balanced upon two feet and stands erect. The tallest apes are obliged to walk upon their feet, as we should walk upon the hands with the great toe standing out from the side of the foot, and the heel is so that it has not the power of supporting the body that our heel has. The great toe is the essential part of the foot in standing or walking. Man is at once placed at a disadvantage when you place him on all fours and make him walk like an ape.

The lecturer then drew a man in that position and said: The head now hangs forward as a great weight, requiring muscles which we do not possess to support it. The curve in the back and the limbs are so arranged that the knee itself touches the ground, our thighs being much longer than the corresponding bones in the upper arm; and we have to raise the thighs so that the feet may touch the ground.

The Professor dwelt upon the effect of walking upon a man's stature, and then exhibited a gradigraph, a simple apparatus, consisting of two hollow tin tubes placed so as to form a right-angle triangle, each tube containing a wooden piston resting on a spring and having attached to the outer end a piece of charcoal. By means of this instrument he showed the variation in height of a person while walking, and also the oscillation of the body from side to side. "I do not" said he "claim for this instrument any wonderful powers, but it is, I think, possible by this means to get a more exact idea of the gaits of different nations. It would certainly be easy to recognise a gait having any distinct characteristics, as, for instance, a stage stride. We know that the French walk different from the Prussians, and the Prussian from the English.—It is possible that this instrument may yet be perfected so as to measure the exact amount of oscillation upward and downward and from side to side. One very curious fact in regard to walking is that one side of the body always tends to outwalk the other side. It is not possible, when the eyes are shut, to walk in a straight line for any length of time.

